# Study on the diversity of mental states and neuroplasticity of the brain during human-machine interaction

**DOI:** 10.3389/fnins.2022.921058

**Published:** 2022-12-07

**Authors:** Teng Zhang, Xiaodong Zhang, Wenjing Zhu, Zhufeng Lu, Yu Wang, Yingjie Zhang

**Affiliations:** ^1^School of Mechanical Engineering, Xi’an Jiaotong University, Xi’an, China; ^2^Shaanxi Key Laboratory of Intelligent Robot, Xi’an Jiaotong University, Xi’an, China

**Keywords:** human-machine collaboration, functional brain network, EEG, mental state diversity, neuroplasticity

## Abstract

**Introduction:**

With the increasing demand for human-machine collaboration systems, more and more attention has been paid to the influence of human factors on the performance and security of the entire system. Especially in high-risk, high-precision, and difficult special tasks (such as space station maintenance tasks, anti-terrorist EOD tasks, surgical robot teleoperation tasks, etc.), there are higher requirements for the operator’s perception and cognitive level. However, as the human brain is a complex and open giant system, the perception ability and cognitive level of the human are dynamically variable, so that it will seriously affect the performance and security of the whole system.

**Methods:**

The method proposed in this paper innovatively explained this phenomenon from two dimensions of brain space and time and attributed the dynamic changes of perception, cognitive level, and operational skills to the mental state diversity and the brain neuroplasticity. In terms of the mental state diversity, the mental states evoked paradigm and the functional brain network analysis method during work were proposed. In terms of neuroplasticity, the cognitive training intervention paradigm and the functional brain network analysis method were proposed. Twenty-six subjects participated in the mental state evoked experiment and the cognitive training intervention experiment.

**Results:**

The results showed that (1) the mental state of the subjects during work had the characteristics of dynamic change, and due to the influence of stimulus conditions and task patterns, the mental state showed diversity. There were significant differences between functional brain networks in different mental states, the information processing efficiency and the mechanism of brain area response had changed significantly. (2) The small-world attributes of the functional brain network of the subjects before and after the cognitive training experiment were significantly different. The brain had adjusted the distribution of information flow and resources, reducing costs and increasing efficiency as a whole. It was demonstrated that the global topology of the cortical connectivity network was reconfigured and neuroplasticity was altered through cognitive training intervention.

**Discussion:**

In summary, this paper revealed that mental state and neuroplasticity could change the information processing efficiency and the response mechanism of brain area, thus causing the change of perception, cognitive level and operational skills, which provided a theoretical basis for studying the relationship between neural information processing and behavior.

## Introduction

In the actual human-machine collaboration environment or daily life when using computers and various other large, medium and small machines (including the operation of aircraft, cars, trains, and boats), human perception, cognitive level and operational skills is in a dynamic change in real-time, which will seriously affect the performance and safety of the system (Zhang et al., 2022). Studies have shown that (1) the high mental workload of the human brain will cause rapid fatigue, reduced flexibility, stress response, increased human error, and frustration, resulting in errors in information acquisition and analysis and decision-making errors, and then lead to decreased performance. Therefore, it is a significant cause of human accidents. However, the too low mental workload will cause waste of human resources and other resources, cause disgust, and also lead to the decline of operational performance (Wilson, 2005). (2) Fatigue causes a decrease in individual feeling, perception and reaction ability, slow thinking, memory loss, inattention, reduced alertness, insensitivity to external environmental stimuli, and insufficient work motivation (Boksem et al., 2005, 2006; Zhang et al., 2019). In addition, fatigue reduces the brain’s ability to process automatic and conflicting information, resulting in reduced cognitive performance (Yang, 2009). Fatigue can weaken the executive function of the frontal lobe of the brain and decrease involuntary attention and selective attention (Boksem and Tops, 2008). Fatigued people are also less likely to correct behavioral errors and show a reduced ability to monitor and regulate behavior (Lorist et al., 2005). In a complex human-machine system, most people and systems are in a special and extreme working environment, coupled with high-intensity workloads, which can easily lead to mental fatigue of operators, resulting in reduced performance and increased errors (Ricci et al., 2007). (3) Emotions affect people’s perception and cognitive level and affect the processes of attention, memory, and reaction (Reeves et al., 1991; Stanley and Larsen, 2021), thereby determining the ability of situational awareness (John and Gross, 2004). In a positive emotional state, people tend to pay more attention to the overall situation of things and can see or remember the main outline of things. In contrast, people are more likely to remember the details of things in a negative emotional state. Moreover, all kinds of emotional experiences can play a role in our decision-making process (Peters et al., 2006). For example, positive emotions play a benign role in the decision-making process of switching between automated driving and manual driving (Du et al., 2020). (4) As vigilance decreases, operational performance decreases (Langner and Eickhoff, 2013). Closely related to vigilance, attention refers to the ability to focus cognitive resources on a particular stimulus (Kivikangas et al., 2011, Hancock, 2013). Insufficient attention level will lead to difficulty in completing tasks, while too great attention or too narrow attention range will affect the progress of subtasks (Frey et al., 2013). The above mental workload, fatigue, emotion, and vigilance are all classified as mental states in this paper. The topology of the functional brain networks underlying each mental state varies significantly in spatial dimensions and is diverse. We call this characteristic of the human brain in the spatial dimension the mental state diversity. which is one of the main reasons for real-time changes in human perception, cognitive level and operational skills.

Moreover, under the same mental state, compared with novices, experienced drivers or operators of special machinery and equipment have higher operational proficiency, which can effectively improve operational performance and reduce the occurrence rate of dangerous accidents caused by human error. The reason is repeated cognitive training intervention can cause changes in brain activity and even long-term changes in brain structure, which is manifested in the improvement of operational skills (Taya et al., 2015). For example, the study found that London taxi drivers with more spatial navigation experience in a complex city have larger gray matter volumes in the hippocampus (Maguire et al., 2006). Professional typists who focus on long-term typing practice have increased gray matter volume in brain regions associated with motor tasks, such as the supplementary motor area, prefrontal cortex, and cerebellum (Cannonieri et al., 2007). Since violinists and other string players use the second to fifth fingers of the left hand to play the strings, this results in a greater representation of the left hand fingers in the primary somatosensory cortex (Elbert et al., 1995). Experiments have also shown that after cognitive training interventions, the connectivity, activation, and reorganization properties of brain regions responsible for corresponding functions can be altered, such as athletes who undergo regular training can not only change their brains at the structural level, but also change the brain processing and activation patterns in the context of sports (Seidel-Marzi and Ragert, 2020). Rehabilitation and intensive training intervention can improve the motor ability of people with related functional disabilities, which is inherently manifested as changes in the neural pattern of activation and reorganization of the ipsilateral or contralateral hemisphere of the brain (Feitosa et al., 2022). We call the changes in brain neural activity and morphology caused by repeated and regular reinforcement training in the time dimension as neuroplasticity, that is, the characteristics of the brain in the time dimension. This is also the second main reason for the changes in human perception, cognitive level and operational skills.

To sum up, two main reasons for the dynamic changes of perception, cognitive level and operational skills in the actual operating environment are the mental state diversity and neuroplasticity which represent two significant properties of the brain in spatial dimension and time dimension, respectively. More specifically, the brain is a complex network consisting of spatially distributed regions dedicated to different functions, and it is proposed that mental states functions emerge from dynamic interactions of several brain areas, not from activation of a single brain region. Because in different mental states, the topological structure of the functional connection network of the human brain shows different forms in space, so the diversity of mental states is the attribute of the human brain in the spatial dimension. Moreover, in the process of evolution, development, and remodeling of living organisms, the strength of synaptic connections between neurons, internal activation of neurons, physical structure, and other aspects are shaped by the constantly changing internal and external environment all the time (Wang, 2020). Neuroplasticity changes can also be triggered in the adult brain through two fundamental processes, learning and cognitive training, although changes in brain structure are thought to be limited to critical periods of development (Draganski and May, 2008). Whether the period is years, months, days, hours, or minutes, such neuroplasticity changes over time are attributes of the brain on the time dimension. In summary, mental state diversity and neuroplasticity can be described as two major properties of the brain in spatial and temporal dimensions, respectively. However, few people have comprehensively studied the dynamic changes in the spatial and temporal dimensions of the human brain during work.

Moreover, with the emergence and development of non-invasive brain function monitoring technologies, such as electroencephalogram (EEG) (Li et al., 2022), magnetoencephalography (MEG) (Baillet, 2017), functional magnetic resonance imaging (fMRI) (Power et al., 2017), the interrelationship between human perception, cognition and performance, systems, and technology can be studied from many perspectives. In terms of mental state, for example, Li et al. (2019) designed a mental arithmetic task to induce mental fatigue in the subjects. Significant differences were found between EEG-based functional brain networks before and after the task, with marked changes in their small-world properties. Ghassemzadeh et al. (2019) proposed that the regulation and improvement of attention can be observed through changes in brain networks. Liu et al. (2019) and Wu et al. (2022), respectively proposed that the use of EEG-based functional brain network features has advantages in recognizing emotions. And Liu et al. (2019) found that the spatiotemporal topology of dynamic functional connectivity shows small-world structure. In terms of neuroplasticity, for example, Romero et al. (2008) trained subjects by designing an alphabet addition task, found that EEG of subjects before and after training was significantly different, and proved that EEG can be used as an electrophysiological marker of skill-related neuroplasticity. Seppanen et al. (2012) also observed neuroplasticity using EEG by designing a music training task. The feature of the study was to demonstrate that short-term (within tens of minutes) training tasks also triggered neuroplasticity in the subjects. In conclusion, EEG-based brain network features can effectively reflect different mental states, and EEG can also be used as an electrophysiological marker for judging neuroplasticity (Thibaut et al., 2017). Human-centered cognitive state monitoring, cognitive enhancement, closed-loop adaptive human-machine interaction and other technologies have become research hotspots. Parasuraman (2011) defined this discipline mainly focused on studying brain and behavior in work as neuroergonomics. Compared with traditional ergonomics, modern ergonomics faces increased system complexity, and the relationship between people and systems is nonlinear and fuzzy. Humans’ ability to understand and simulate complex human-system interactions at work depends on their knowledge of the complexity of neural information processing, rather than solely on measures of workers’ explicit behavior and subjective perceptions. Therefore, this paper uses EEG and functional brain network techniques to demonstrate that changes in mental state and neuroplasticity occur during human-machine collaboration by designing a typical mental state evoked paradigm and cognitive training intervention paradigm. The functional brain network topology formed by the interaction of various functional areas in the spatial and temporal dimensions of the brain is analyzed, which may help people to further study the information processing mechanism and mental expression mechanism in the brain. The reasons for the mental states diversity and neuroplasticity changes induced during work process are explored to provide theoretical support for behavioral approaches to improving performance. Further, by analyzing the neurobiological mechanism behind it, it can promote the improvement of neural enhancement technology, adaptive automation technology and enhanced cognition technology, which is of great significance for the development of brain enhancement systems for the field of human-machine interaction.

This article is organized as follows: in section “Materials and methods”, the typical mental state evoked paradigm during work and the cognitive training intervention paradigm are designed, and the functional brain network analysis method is developed. Then the participant and experimental materials are described. In section “Results,” (1) the differences between the functional brain networks of typical mental states, the transmission efficiency and the response mechanism of the related brain regions are analyzed, and the characteristics of the dynamic changes of the mental state of the human brain during work are revealed. (2) The differences in the small-world topological properties of the user’s brain network before and after cognitive training caused by neuroplasticity and the neurobiological mechanisms behind it are analyzed. Section “Discussions” discusses the results, their implications, limitations, and future research efforts. Finally, section “Conclusion” summarizes the main conclusions of this paper.

## Materials and methods

### Analysis method for mental state diversity

#### Description of mental state evoked system

**FIGURE 1:**
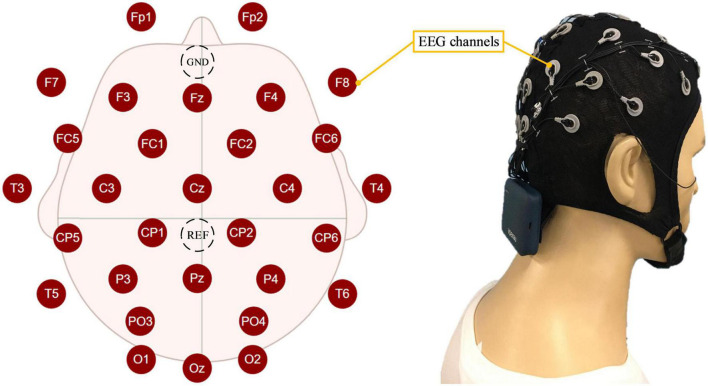
The distribution of electroencephalogram (EEG) electrodes and how the participant wore them.

**FIGURE 2:**
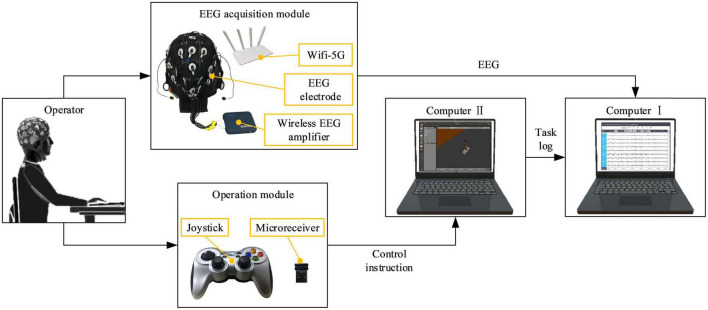
An overview of the mental state evoked system.

The proposed mental state evoked system mainly included the EEG acquisition module, the computer, and the operation module. The EEG acquisition module was mainly responsible for acquiring, amplifying, and transmitting EEG to the computer. The EEG-W32 model equipment produced by Neuracle Technology Co., LTD. was used, the sampling frequency was 1000 Hz, and the communication method was WiFi. This device consisted of 30 measuring electrodes, one reference electrode (REF) and one ground electrode (GND). The impedance level of all measuring electrodes were kept below 10 kΩ in each experiment. Electrode distribution conformed to international 10–20 standards (Figure 1). The computer module consisted of two computers. Computer I was used to record and analyzed EEG, and computer II was used to run virtual tasks. Microprocessor with Intel (R) Core (TM) i7-10710 CPU and i5-4590 CPU were employed in the computer I and computer II, respectively. This module used MATLAB and Robot Operating System melodic. The operation module was used to realize the human-machine interaction function, and it mainly included the joystick. The F710 wireless joystick developed by Logitech (China) Technology Co., LTD. was used, and it adopted 2.4 GHz wireless technology. The overview of the system is illustrated in Figure 2, when the operator played games through the joystick, the EEG was collected and transmitted to the computer in real-time.

#### Subjects and mental state evoked experimental procedure

Twenty-six healthy subjects participated in the experiment (the age range was 20–35, seven female and 19 male subjects), of which 13 participants participated in the mental state evoked experiment, and the remaining 13 participants participated in the cognitive training intervention experiment. All participants reported normal or corrected-to-normal vision and had no previous experience with the mental state evoked and cognitive training interventions systems. Written informed consent was obtained from each participant before the experiment. The Institutional Review Board of Xi’an Jiaotong University approved the proposed experiment, and all experiments were conducted following the Declaration of Helsinki.

**FIGURE 3:**
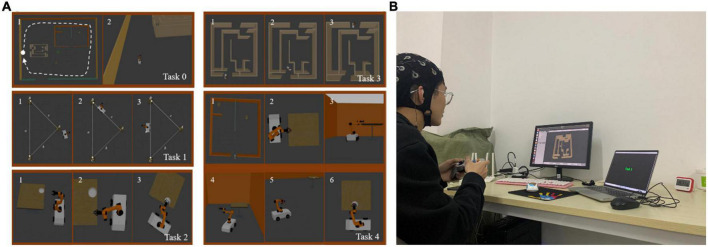
**(A)** The task interface of a typical mental state evoked system. **(B)** The experimental scenario.

In this experiment, subjects were required to operate a virtual robot to perform virtual manipulation tasks. By setting five different operation tasks and adding corresponding stimulation conditions during the operation, various typical mental states (there are mainly resting state, fatigue state, attentive state, inattentive state, positive state, and negative state) were induced. The EEG acquisition module collected EEG in real time and made a marking process. The mental state label was obtained according to the subjective evaluation of the subjects. Among them, the interface of five kinds of operation tasks is shown in Figure 3A, and the specific requirements of each task are as follows: in task 0, the subjects need to control the robot to move in a clockwise circle in a rectangular room, without restricting the robot’s movement trajectory, as shown by the dotted line in the figure. In task 1, the subjects need to control the robot to walk along the prescribed trajectory, namely the sides a and c of the triangle, as shown by the thin solid lines in the figure. In task 2, the subjects were required to control the robot to grab the small cube on the table, and then place the cube in the white tray. Task 3 is a time-limited task in which the subjects need to control the robot to walk out of the maze within 1 min. In task 4, compared to task 2, the small cube was replaced by a cylinder bottle, and the tray was placed on a table in another room. The subjects were asked to control the robot to grab the cylindrical bottle and move it to another room, placing the cylindrical bottle in a white tray. The difficulty level of task 0 to task 4 is from 1 to 5, the higher the number, the higher the difficulty. The stimulation conditions in the experiment mainly include (1) the operation interface is blocked or the background is blurred, so that the subjects can improve their attention. (2) Repeatedly perform a single task for a long time, so that the subjects lose concentration. (3) Performing complex tasks with high mental workload for a long time, thus making the subjects mentally fatigued. (4) Prompts for correct operation and reward prompts appear, thus inducing a positive state of the subjects. (5) Prompts of operation errors and task countdowns appear, thereby inducing a negative state of the subjects.

The correspondence between each task and the mental state expected to be evoked is as follows: Task 0 had the lowest difficulty level and had no time requirement, so it was used to induce the subject’s inattentive state at the end of the task; The difficulty level of Task 1 was slightly higher than that of Task 0, so it was used to induce the attentive state of the subjects; The difficulty level of Task 2 was medium (level 3). According to the real-time performance of the subjects when performing the task, with the stimulation conditions preset in the experiment, it could be used to induce positive and negative states of the subjects; Task 3 set the condition of time limit, which could increase the subject’s concentration or sense of urgency, so it was used to induce the subject’s attentive or tension state; Task 4 had the highest difficulty level and could induce fatigue in the subjects by performing difficult tasks for a long time.

The experimental steps are as follows: firstly, the subjects sit quietly in front of the computer screen wearing an EEG cap, so that their hands can comfortably control the handle, and the experimental scene is shown in Figure 3B. Before the experiment, the subjects were asked to be familiar with the experimental requirements and the experimental procedure. Then, the experiment officially started, and each subject was asked to perform two rounds of the task. The execution sequence of each round is Task 1 → Task 2 → Task 3 → Task 0 → Task 4. Stimulation conditions were preset, and when a specific task was performed, a specific stimulus condition would appear, in order to induced a related mental state. When the subjects performed the task, the EEG acquisition module would collect EEG in real time and do marking processing. Each time the EEG data was recorded, the subjects were required to complete a subjective evaluation. The total experimental duration of each subject was 2 h.

**FIGURE 4:**
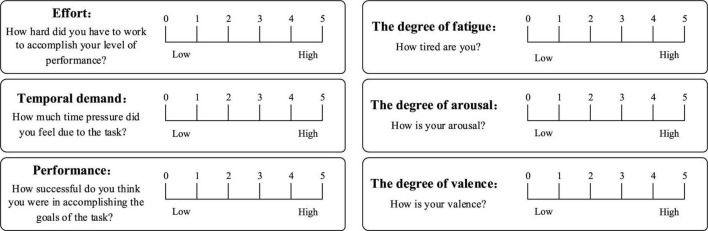
Subjective evaluation scale for typical mental states.

In order to make the subjects complete the subjective evaluation simply, intuitively and efficiently, we designed a subjective evaluation scale for the operator’s mental state based on the SAM scale (Balasubramanian et al., 2018) and the NASA-TLX scale (Harry et al., 2021; Figure 4). It included six evaluation indicators, and the degree of each evaluation indicator was divided into five grades, and the combination of multiple evaluation indicators was used to judge the type of mental state. The corresponding relationship between the index parameters in the subjective evaluation scale and the typical mental state is shown in Table 1, where “/” stands for unlimited.

**TABLE 1:** Correspondence between the indicators parameters and typical mental states in the subjective evaluation scale.

Indicators of subjective evaluation scale						Typical mental state
Effort	Temporal demand	Performance	The degree of fatigue	Arousal	Valence	
/	Medium	/	/	≥Medium	Medium	Attentive state
Low	Low	/	/	Low	Low	Inattentive state
/	/	/	High	Low	Medium	Fatigue state
/	/	High	/	≥Medium	High	Positive state
/	/	Low	/	≥Medium	Low	Negative state

#### Data processing

The data processing mainly includes four steps. Step 1: filter noise, remove baseline and ocular electrical signals from the collected EEG, detailed steps can be found in our previous study (Zhang et al., 2021). Step 2: on the whole-brain scale, the location of the 30 channels in the EEG acquisition device is used as the node of the brain network. Then, based on the EEG data, calculate the phase lag index (*PLI*) between each channel at low frequency band (that is, the frequency bands of theta and alpha rhythm waves are 4.0∼13.0 Hz) and high frequency band (that is, the frequency band of the beta rhythm wave is 14.0∼30.0 Hz), respectively, since the delta rhythm wave appears only during sleep, deep anesthesia, hypoxia and organic brain lesions, this rhythm wave is not considered in this paper. Step 3: according to the size of the *PLI*, the functional connections between nodes in the brain network are measured, and a series of threshold correlation matrices are generated by setting parameters such as connection thresholds to describe the functional brain network. Step 4: the small-world network attribute parameters of functional brain networks are calculated according to graph theory, and the statistical differences between the indicators of various typical mental states are analyzed by using the Two-sample *T*-test method (Xu et al., 2017). The following is a detailed introduction.

The brain is an extremely complex network, which is interrelated on different temporal and spatial scales. By studying the brain’s neurons and the connections between them, we can understand the coordination between brain regions and the functional cognitive principles of the brain. A large number of research results have proved that the brain is neither a completely random network nor a completely regular network, but an “economic” small-world topology network (Sun et al., 2014). The so-called small-word network refers to that it has a small characteristic path length *L* and a large clustering coefficient *C*. In general, the clustering coefficient *C*_*i*_ of a node *i* with degree *K*_*i*_ is defined as the ratio of the number of existing edges (#*_*i*_*) between neighbors of *i* (a node *j* is called a neighbor if *A*_*ij*_ = 1) and the maximum possible number of edges. This can be formalized as (Ponten et al., 2010),


(1) $$C_{i}=\frac{2{\#}_{i}}{K_{i}\left(K_{i}-1\right)}=\frac{1}{K_{i}\left(K_{i}-1\right)}\sum_{j=1}^{N}\sum_{o=1}^{N}A_{ij}A_{io}A_{oj}$$

where *C*_*i*_ is an index of local structure, which has been interpreted as a measure of resilience to random error (in case node *i* is lost its neighbors remain connected if *C*_*i*_ is large). *C* is the mean clustering coefficient of the graph. That is,


(2) $$C=\frac{1}{N}\sum_{i=1}^{N}C_{i}$$

The path length *L*_*ij*_ between two nodes *i* and *j* is the minimal number of edges that have to be passed to connect *i* and *j*. The mean shortest path length *L* of a graph is the mean *L*_*ij*_ between all possible pairs of nodes. We note that using the harmonic mean allows for inclusion of isolated nodes (for which *L*_*ij*_ → ∞). That is,


(3) $$L=\frac{1}{N\left(N-1\right)}\sum_{i\neq j}L_{ij}$$

The small-world coefficient σ is an indicator that indicates whether a network has the small-world topological attribute. σ can be calculated according to *C* and *L*. That is,


(4) $$\begin{array}{c} \gamma =C/C_{\text{random}}\\ \lambda =L/L_{\text{random}}\\ \sigma =\gamma /\lambda \end{array}\}$$

*C*_random_ and *L*_random_ are the clustering coefficient and characteristic path length of the random network, respectively. If σ > 1, it means that the network has the small-world topology property, otherwise it does not have the small-world topology property. Another commonly used measure is network global efficiency *E*_*g*_, which is the average of the reciprocal of *L*_*ij*_. Both indicators *E*_*g*_ and *L*_*ij*_ can better measure the global information processing and transmission capabilities of the network, as well as the degree of integration of the network. This can be formalized as,


(5) $$E_{\text{g}}=\frac{1}{N\left(N-1\right)}\sum_{i\neq j}\frac{1}{L_{ij}}$$

In addition, network local efficiency *E*_loc_ is the average of all node local efficiencies *NE*_loc_, which can measure the degree of differentiation of the network, so that it can effectively characterize the local characteristics of the brain network. The parameters introduced above are global attribute parameters. This paper also uses a local attribute parameter called *NE*_loc_, which characterizes the efficiency of parallel information transmission of the node in the network. This can be formalized as,


(6) $$NE_{\text{loc}}\left(i\right)=\frac{1}{N_{G_{i}}\left(N_{G_{i}}-1\right)}\sum_{j\neq k}\frac{1}{L_{jk}}$$

where *G*_*i*_ refers to the subgraph formed by the adjacent nodes of node *i*, and *L*_*jk*_ represents the length of the shortest path between nodes *j* and *k*.

When we construct a functional brain network, the definition of the edges between nodes adopts the *PLI*, which can better avoid the effect of volume conductors. It is an index to detect the asymmetry of phase difference distribution between two signals, it can reflect the consistency of phase advance or lag of one signal relative to another, and it is an effective estimate of phase synchronization. The biggest advantage of *PLI* is that it is insensitive to the volume conductor effect of signals (Winter et al., 2007) and can only focus on the coupling relationship between signals (Stam et al., 2009). The specific calculation process is as follows, assuming that *φ_*n*_* and *φ_*m*_* are the phases of two time series and Δφ is the phase difference between them, then the phase synchronization index between time series *n* and time series *m* (*n, m* is an integer) is defined as the following formula,


(7) $$\left|\Delta \varphi_{nm}^{PQ}\right|=\left|P\varphi_{n}-Q\varphi_{m}\right|<\text{constant}$$

we qualified *P* = *Q* = 1. To calculate phase synchronization, we need to know the instantaneous phase of the two signals. It can be obtained by the Hilbert transformation operation of the analytic signal ψ(*t*). The ψ(*t*) can be obtained from a real time series *S*(*t*) and its Hilbert transform $\tilde{S}\left(t\right)$, as shown in formula,


(8) $$\begin{array}{c} \psi \left(t\right)=S\left(t\right)+\text{i}\tilde{S}\left(t\right)=A\left(t\right)e^{\text{i}\varphi \left(t\right)}\\ \tilde{S}\left(t\right)=\pi^{-1}\int_{-\infty }^{\infty }\frac{S\left(\tau \right)}{t-\tau }d\tau \\ A\left(t\right)=\sqrt{S{\left(t\right)}^{2}+\tilde{S}{\left(t\right)}^{2}}\\ \varphi \left(t\right)=\arctan\frac{\tilde{S}\left(t\right)}{S\left(t\right)} \end{array}\}$$

where *A*(*t*) represents the instantaneous amplitude and φ(*t*) represents the instantaneous phase. *PLI* estimates the insensitive phase synchronization from the same source by calculating the asymmetry of the phase difference distribution law, as shown in formula,


(9) $$PLI=\left|\left\langle sign\left(\Delta \varphi \left(t\right)\right)\right\rangle \right|=\left|\frac{1}{N}\sum_{n=1}^{N}sign\left(\Delta \varphi \left(t_{n}\right)\right)\right|$$

where *sign* is a sign function. The variation range of *PLI* is 0–1. When *PLI* is 0, it indicates that there is no coupling relationship, and when *PLI* is 1, it indicates that there is a perfect phase locking relationship.

### Analysis method for neuroplasticity of the brain

#### Description of cognitive training interventions system

Similar to the mental state evoked system mentioned above, cognitive training interventions system mainly includes three modules: the EEG acquisition module, the computer, and the operation module. The difference is that the operation module is composed of mouse and keyboard, because the virtual task has changed.

#### Cognitive training interventions experimental procedure

**FIGURE 5:**
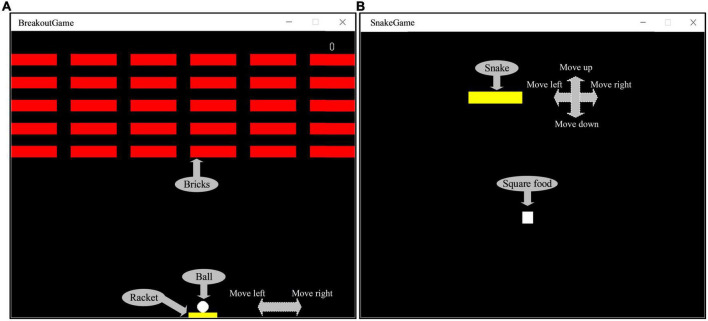
**(A)** The Breakout game interface. **(B)** The Snake game interface.

**FIGURE 6:**
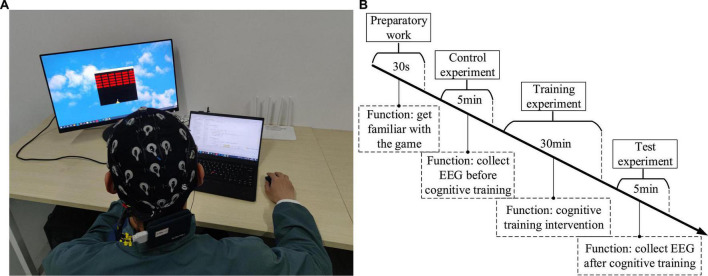
**(A)** The experimental scenario. **(B)** The experimental steps.

Studies have found that one of the best approach is to use video games to restore the brain or improve functional plasticity (Bavelier and Green, 2003; Ballesteros et al., 2015; Valentin, 2017). Because of its ease of use and the numerous potential applications, the cognitive training has attracted substantial public attention, and a lot of computer software for “brain training” are available on web, PCs or smartphones. Hence, two kinds of game were designed in this paper. One was Breakout game and the other was Snake game, which were tweaked from the original Atari games (Bevilacqua et al., 2016; Cuccu et al., 2021). Figure 5A shows that the operator can use the mouse to control the racket to hit the ball and make it hit bricks. The more bricks it hits, the higher the score; on the contrary, the fewer bricks it hits or the game fails, the lower the score. The way to calculate the score is as follows: 1 point for each brick hit, and the racket successfully hits the ball five times in a row, the speed of the ball will increase by one level, at this time, 2 points for each hit of a brick. By analogy, the higher the speed rating, the higher the score for each brick hit. The scoring stops when the game is cleared or failed. Figure 5B shows that the operator can control snake’s direction by using the arrow keys on the keyboard, so that snake can eat the square. The more the snake eats, the higher the score; on the contrary, the less the snake eats or game fails, the lower the score. The way to calculate the score is as follows: 1 point for each square eaten, and if the snake eats two squares in a row, the speed of the snake will increase by one level, at this time, 2 points will be awarded for each square eaten. By analogy, the higher the speed level, the higher the score for eating a square. The scoring stops when the game is cleared or failed. The computer records the game task score for each session to evaluate the quality of the control. The game task score can be used to comprehensively evaluate the operation accuracy and reaction time in the behavioral data. If the subject’s operation accuracy is poor, it is easy to cause the game to fail, resulting in a low score. In addition, if the subject’s reaction time is slow, when the speed level increases, the game will also fail and the score will be lower. Conversely, if both the operation accuracy and reaction time have high levels, the game score will be high. The whole experiment process was mainly divided into three stages: control experiment stage, training experiment stage and test experiment stage. The control experiment stage was set before the training experiment stage, while the test experiment stage was set after the training experiment stage, in order to compare the difference between the control experiment and the test experiment results. In the control experiment stage, participants were required to wear an EEG cap, sit quietly in front of the computer screen, and allow their hands to comfortably control the mouse or keyboard, and perform game tasks for 5 min, then the score of the game task was recorded, and the EEG were collected during the 5 min. Participants can choose to rest for 30 s between each game session. Similarly, the same setup was carried out in the test experiment phase. In the training experiment stage, the subjects were required to perform game tasks for about 30 min. If the game was cleared, the experiment could be ended in advance. This stage was the cognitive intervention training stage. Before the control experiment, 30 s of experiment familiarity was set up. In order to control the variables in the control and test experiment stage, we had made efforts in three aspects: (1) In terms of EEG acquisition equipment, the secondary wearing of the EEG cap during the experiment was avoided. Since the re-wearing of the EEG cap will affect the collected signals, we controlled the duration of the experiment within a reasonable range to avoid the situation of wearing the EEG cap twice during the experiment, and ensure that the electrode position and resistance value of the EEG cap remain unchanged. (2) In terms of the subject’s mental state, the different mental states of the subjects in the two experimental stages were avoided. The duration of the training experiment stage was no more than 30 min, the task difficulty was moderate, and a rest session was set to avoid the subjects from mental fatigue to the greatest extent. And in the two experimental stages, a subjective questionnaire survey was conducted on the mental state of the subjects, and if there was a difference in the mental state, the data was excluded. (3) The game task parameters of the two experimental stages were set the same, so that the subjects performed the same game tasks in the control and test experimental stages. Figure 6 shows the experimental scenario and the experimental steps. Other requirements are the same as described above, and the data processing steps are also the same as described above.

## Results

### Result for mental state diversity

In order to analyze the differences of functional brain networks in various typical mental states and the neurobiological mechanisms behind them, three pairs of typical mental states were selected for pairwise comparative analysis: resting state and fatigued state, attentive state and inattentive state, and positive state and negative state. We calculated the *PLI* matrix of each mental state induced by all subjects during the task and calculated its mean value. Then the functional brain network diagram and connection matrix diagram of each typical mental state were drawn. Firstly, the differences among the three pairs of typical mental states are qualitatively analyzed.

#### Resting state and fatigued state

**FIGURE 7:**
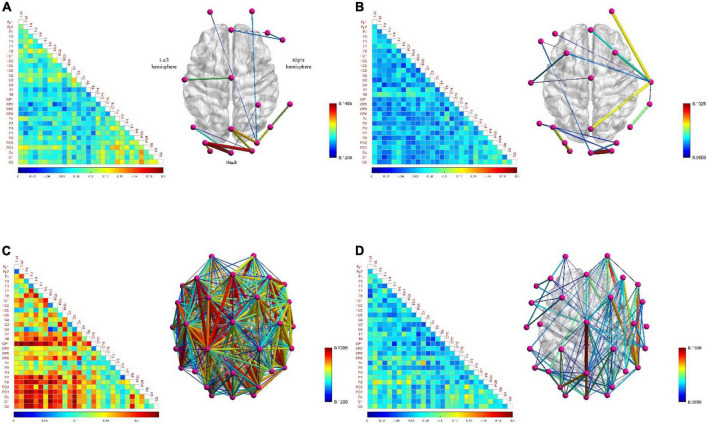
The functional brain networks and connectivity matrices in resting and fatigued states. The perspective of the brain network map is a top view, with the left hemisphere of the brain on the left, and the occipital lobe area below. The lower limit of the connection threshold is manually adjusted to better display the characteristics, and the upper limit is the highest value of the network connection strength. The matrix diagram shows the lower triangular area of 30 channels, where the channels are arranged from top to bottom in order from the front (frontal lobe) to the back (occipital lobe) in the brain area, the same below. **(A)** Represents the situation when the electroencephalogram (EEG) is at low frequency in the resting state. **(B)** Represents the situation when the EEG is at high frequency in the resting state. **(C)** Represents the situation when the EEG is at low frequency in the fatigue state. **(D)** Represents the situation when the EEG is at high frequency in the fatigue state.

Figures 7A,C show that the functional brain network connectivity density of low-frequency EEG increased abruptly when the subjects moved from the resting state to the fatigued state. One of the reasons for this phenomenon may be that when adults are fatigued, the slow waves in the EEG gradually increase, while the fast waves gradually decrease (Borghini et al., 2014), so that the functional brain network connection density of low-frequency EEG under fatigue conditions becomes denser. However, Figures 7B,D show that, relative to the resting state, the connectivity density of the functional brain network for high-frequency EEG in the fatigued state does not decrease but increases, although the increase is much smaller than in the case of low-frequency EEG. The reason is that the subjects in the fatigued state are in the process of work and need to mobilize brain resources to maintain a high level of perception and cognition, so as to effectively perform game tasks. Therefore, this reflects that the functional brain network connection density of both low-frequency and high-frequency EEG increased in the fatigue state compared with the resting state during work, and the connectivity density of the brain network with low-frequency EEG was increased to a higher degree due to further influence by the fatigue state. This phenomenon shows that the fatigue state during work is significantly different from that during non-work. In addition, it is interesting that the functional brain network of high-frequency EEG has lower connectivity density in the left hemisphere than in the right hemisphere under fatigue state, showing an asymmetric connectivity pattern, as shown in Figure 7D. This indicates that the processing response of the brain area is biased to the right in the fatigue state, and this phenomenon has also been confirmed in Sun et al. (2014).

**FIGURE 8:**
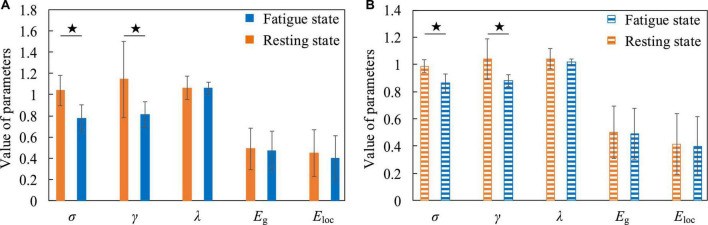
The contrasting histograms of five global attribute parameters of functional brain networks in resting and fatigued states. “★” indicates statistical difference, the same as below. **(A)** The case when the electroencephalogram (EEG) is at low frequency. **(B)** The case when the EEG is at high frequency.

The quantitative analysis of resting and fatigued states was carried out below. We calculated the average value of the parameters of the small-world network whose sparsity ranged from 5 to 40% of the functional brain network of all subjects, and used the Two-Sample *T*-test method to statistically analyze the differences between each pair of parameters (the settings for the quantitative analysis below are the same). We compared σ, γ, λ, *E*_*g*_, and *E*_loc_ five small-world network parameters. Figure 8 shows that compared to the resting state, the σ in the fatigued state is significantly reduced (low frequency, t = 3.873, *p* = 0.002, effect size of Cohen’s d = 1.937; high frequency, t = 4.315, *p* = 0.001, effect size of Cohen’s d = 2.158), indicating that the “economy” of the functional brain network in the fatigued state decreases, and the brain needs to expend more resources to maintain a high level of perception and cognition. At the same time, the γ in the fatigue state is significantly reduced (low frequency, t = 2.486, *p* = 0.026, effect size of Cohen’s d = 1.243; high frequency, t = 2.919, *p* = 0.011, effect size of Cohen’s d = 1.459). It indicates that in the process of brain processing information flow, the information processing ability of the local brain network under fatigue decreases, leading to the reduction of cluster characteristics of functional differentiation among the called parts of the brain regions, and the information connectivity and collaborative processing between the local brain regions become weak.

#### Attentive state and inattentive state

**FIGURE 9:**
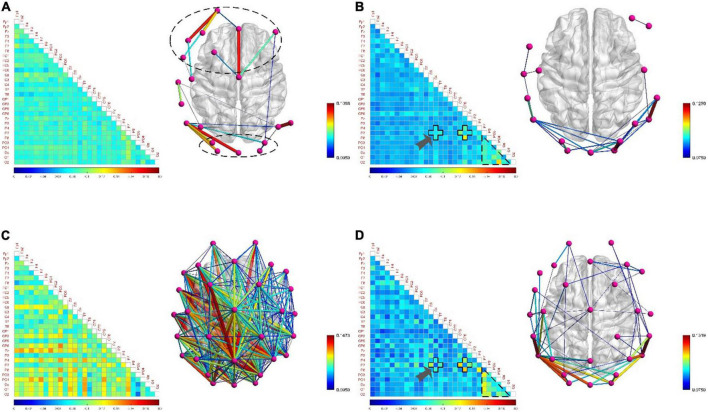
The functional brain networks and connectivity matrices in attentive and inattentive states. **(A)** The situation when the electroencephalogram (EEG) is at low frequency in the attentive state. **(B)** The situation when the EEG is at high frequency in the attentive state. **(C)** The situation when the EEG is at low frequency in the inattentive state. **(D)** The situation when the EEG is at high frequency in the inattentive state.

Figure 9A shows that the densely connected areas of the functional brain network of the low-frequency EEG are mostly concentrated near the frontal and occipital lobes when the subjects are in attentive state. This is because the frontal lobe can enhance the excitability of a certain part of the brain area, so that the related stimuli can be noticed, while inhibiting other brain areas, so it can manage attention, concentrate and maintain a high degree of attention. At the same time, the brain regions involved in the regulation of attention also include the inferior parietal cortex, the superior temporal cortex, and the occipital lobe. This phenomenon has also confirmed in the study of Rosenberg et al. (2016). The connectivity density of functional brain networks for low-frequency EEG increased slightly when attention is decreased (Figure 9C). Comparing Figures 7A,C, 9A,C, it is found that the decrease in concentration and arousal have the same phenomenon in the functional brain network of low-frequency EEG, indicating that there is a certain correlation between attention and arousal. This corroborates the theory that decreased attention is attributed to decreased physiological arousal (Luna et al., 2021). However, the difference degree is much smaller than the difference degree between the fatigue and the resting state, which shows that there is also the difference between the decline of attention and the decline of arousal from another perspective. This may confirm the resource depletion theory proposed by Warm et al. (2008) from the side, that is, the decrease in attention is caused by the lack of replenishment of information processing resources in continuous tasks.

Besides, when the EEG was at high frequency, the degree of difference in connection density between functional brain networks in the attentive and inattentive states was not significant, and the connection patterns shared the same characteristics. For example, in both states, the connection density in the occipital lobe region is high, and the local connection patterns on the P7 channel have the same characteristics, as shown by the arrows in Figures 9B,D. The reason may be that the connectivity properties of the functional brain network of high-frequency EEG are mainly task-dominated, that is, regardless of whether the subject is attentive or inattentive state, when performing the same task, the brain maintains the appropriate level of perception and cognition, as well as the level of limb control and decision-making. The activation degree of the brain regions responsible for related functions and the allocation pattern of brain resources are basically consistent under the influence of the same task, thus showing the same connection characteristics on the functional brain network. This shows that compared with low-frequency EEG, high-frequency EEG contains less mental state information, which is mainly affected by the mobilization of brain resources, the activation level of brain regions and the way of information transmission during work. This phenomenon is also found between positive and negative states, as shown in Figures 11B,D, so the above inference can be generalized to the case of multiple mental states. This highlights another difference between mental states during work and non-work, and provides theoretical support for the detection and identification of mental states during work.

**FIGURE 10:**
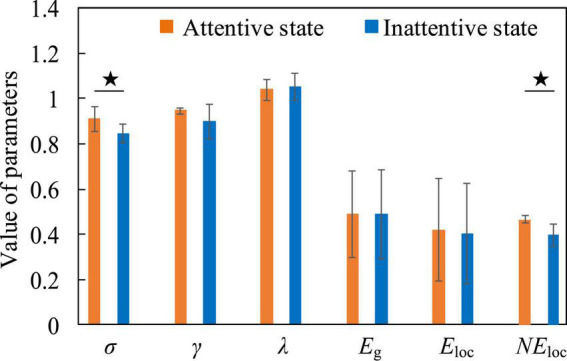
The contrast histograms of five global attribute parameters and one local attribute parameter of the functional brain network in the attentive and inattentive states.

**FIGURE 11:**
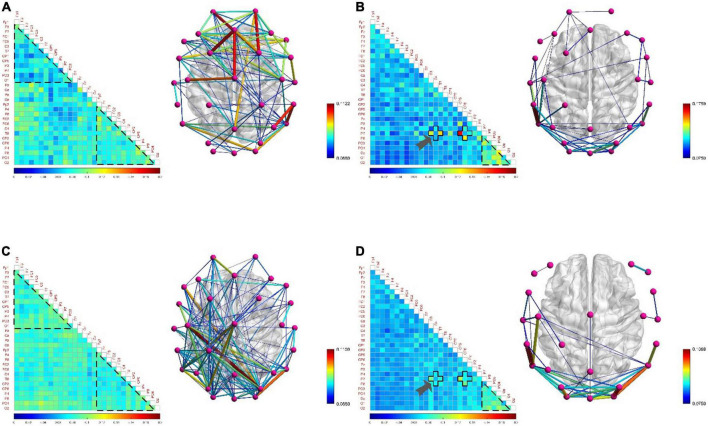
The functional brain networks and connectivity matrices in positive and negative states. **(A)** The situation when the electroencephalogram (EEG) is at low frequency in the positive state. In order to observe the difference between the left and right hemispheres from the matrix diagram, the channels in the matrix diagram are arranged in the order of left hemisphere, central axis, and right hemisphere from top to bottom, and other settings are the same as before. **(B)** The situation when the EEG is at high frequency in the positive state. **(C)** The situation when the EEG is at low frequency in the negative state. The channels in the matrix diagram are arranged in the order of left hemisphere, central axis, and right hemisphere from top to bottom, and other settings are the same as before. **(D)** The situation when the EEG is at high frequency in the negative state.

For attentive and inattentive states, we compared five global attribute parameters and one local attribute parameter of functional brain networks, namely σ, γ, λ, *E*_*g*_, *E*_loc0_, and *NE*_loc_. Figure 10 shows that when the EEG is at a low frequency, compared with the inattentive state, the σ in the attentive state is significantly increased (t = 2.578, *p* = 0.022, effect size of Cohen’s d = 1.289), indicating that maintaining concentration during work improves the “economy” of the small-world attribute of the brain network. The brain optimizes the allocation of resources to maintain high levels of perception and cognition associated with work tasks, thereby making the utilization of brain resources more efficient and the allocation of resources more concentrated. At the same time, the *NE*_loc_ in the occipital lobe area also increased significantly in the attentive state (t = 2.991, *p* = 0.017, effect size of Cohen’s d = 1.892), which proved that the occipital lobe area played a positive role in improving the subjects’ attention during work. Besides, no significant differences were found in the functional brain network of high-frequency EEG, further supporting the speculation that the connectivity properties of the brain network of high-frequency EEG are mainly task-dominated.

#### Positive state and negative state

Figure 11 shows no significant difference and correlation in the connection density of functional brain networks in positive and negative states. The reason may be that the approach motivation system promotes the generation of positive states, while the avoidance motivation system promotes the generation of negative states. Since the approach motivation system and the avoidance motivation system are driven by two independent neural circuits, and require the coordinated activities of different levels of the nervous system and different brain regions to complete, this may be one of the reasons for this phenomenon (Peng, 2019). In addition, when the brain is in a positive state, the connection density of the functional brain network of the low-frequency EEG in the right hemisphere is denser, and that in the left hemisphere is sparser, as shown in Figure 11A; on the contrary, when the brain is in a negative state, the connection density of the functional brain network in the left hemisphere is denser, and that in the right hemisphere is sparse, as shown in Figure 11C, showing the asymmetry and difference between the left and right hemispheres. The underlying reason may be that the positive state increases the activation of the left hemisphere cortex, and the negative state increases the activation of the right hemisphere cortex (Peng, 2019), resulting in the difference between the left and right hemispheres in processing positive and negative states. Moreover, the functional brain network of high-frequency EEG not only did not reveal obvious asymmetries and differences between the left and right hemispheres, but also the connectivity patterns shared the same characteristics. For example, in both states, the connection density in the occipital lobe region is high, and the local connection patterns on the P7 channel have the same characteristics, as shown by the arrows in Figures 11B,D, which proves the above inference.

**FIGURE 12:**
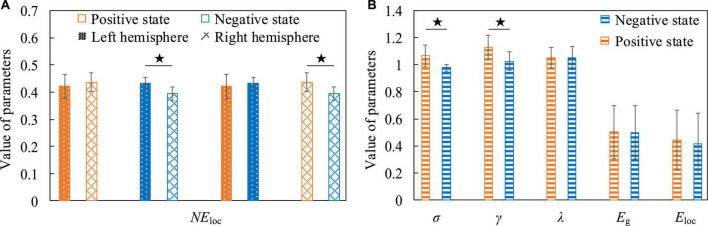
The contrast histograms of five global attribute parameters and one local attribute parameter of the functional brain network during positive and negative states. **(A)** The *NE*_loc_ in different situations when the electroencephalogram (EEG) is at low frequency. **(B)** Five global attribute parameters in two different states when the EEG is at high frequency.

For positive and negative states, we also compared five global attribute parameters and one local attribute parameter of functional brain networks, namely σ, γ, λ, *E*_*g*_, *E*_loc_, and *NE*_loc_. There was no significant difference in the global properties of functional brain networks in positive and negative states when EEG was at low frequency. However, there were significant differences in *NE*_loc_ between the left and right hemispheres of the brain. In Figure 12A, the first group of histograms represent the *NE*_loc_ of the left and right hemispheres in the positive state, respectively. The second group represents the *NE*_loc_ of the left and right hemispheres in the negative state, respectively. The third group represents the *NE*_loc_ of the left hemisphere in positive and negative states, respectively. The last group represents the *NE*_loc_ of the right hemisphere in positive and negative states, respectively. In the negative state, the *NE*_loc_ in the frontal, central and temporal regions of the left hemisphere (channels are F3, F7, FC1, FC5, C3, and T7) are significantly higher than those in the right hemisphere (channels are F4, F8, FC2, FC6, C4, and T8), indicating that the local information transmission efficiency of the nodes in the left hemisphere was significantly increased in the negative state (t = 2.896, *p* = 0.016, effect size of Cohen’s d = 1.672). At the same time, compared with the negative state, the *NE*_loc_ in the frontal, central and temporal regions of the right hemisphere increased significantly in the positive state (t = 2.413, *p* = 0.036, effect size of Cohen’s d = 1.393), indicating that the local information transmission efficiency of the nodes in the right hemisphere was significantly increased in the positive state. It should be noted that previous studies have concluded that positive states increase the activation of the left hemisphere cortex, and negative states increase the activation of the right hemisphere cortex **(Peng, 2019)**, so it can be concluded that the local efficiency of the node is not positively correlated with the activation degree of the cortex. The common conclusion that can be drawn is that the brain has left and right hemisphere differences in processing positive and negative states. Figure 12B shows that σ is significantly higher in the positive state than in the negative state (t = 2.953, *p* = 0.010, effect size of Cohen’s d = 1.477), it shows that the positive state effectively improves the “economy” of the brain’s small-world network and optimizes the allocation of resources, so that the brain can more efficiently maintain the level of perception and cognition required for work. At the same time, the γ in the positive state is significantly elevated (t = 2.613, *p* = 0.020, effect size of Cohen’s d = 1.306). It indicates that in the process of brain processing information flow, the information processing ability of the local brain network under positive state increases, leading to the enhancement of cluster characteristics of functional differentiation among the called parts of the brain regions, and the information connectivity and collaborative processing between the local brain regions become stronger. This may explain why subjects have higher quality of work when they are in a positive state.

### Result for neuroplasticity of the brain

**FIGURE 13:**
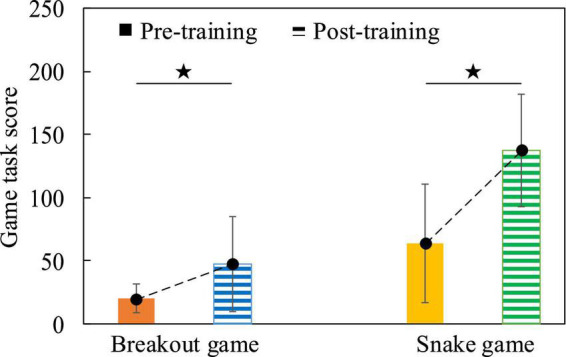
The comparison histogram of the scores of the game tasks before and after the cognitive intervention training, the dotted line indicates the change trend.

Firstly, the behavioral data results of the subjects before and after cognitive intervention training were analyzed. We calculated the average value of the game task scores of all subjects before and after training, and used the Two-Sample *T*-test method to statistically analyze the differences in game task scores. Figure 13 shows that in both game tasks, the game task scores obtained by subjects after training were significantly higher than those obtained before training, (Breakout game, t = 2.501, *p* = 0.020, effect size of Cohen’s d = 0.981; Snake game, t = 4.097, *p* < 0.001, effect size of Cohen’s d = 1.607), indicating that after the cognitive training intervention, the behavioral data results of the subjects changed, and the operational skills were improved.

**FIGURE 14:**
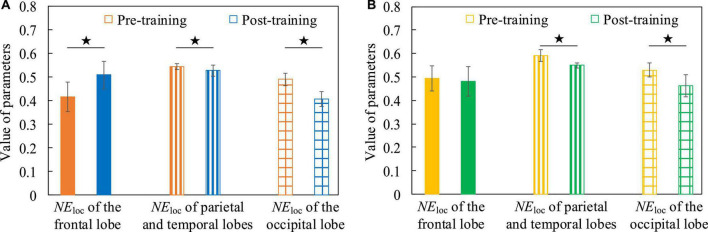
The *NE*_loc_ contrast histograms of functional brain networks in both pre-training and post-training conditions. **(A)** The Breakout game task. **(B)** The Snake game task.

In order to verify the changes of neuroplasticity after cognitive training intervention and analyze the underlying neurobiological mechanisms, we analyzed the global and local attributes of functional brain networks before and after training, and there was no significant difference in global attributes, but significant differences in local attributes, mainly on *NE*_loc_. The mean values of *NE*_loc_ in frontal lobe, parietal lobe, temporal lobe and occipital lobe of all subjects participating in this experiment were calculated, respectively, and the difference between each pair of parameters was statistically analyzed by Two-Sample *T*-test method. In the Breakout game task, Figure 14A shows that when the EEG is at a low frequency, *NE*_loc_ in the frontal lobe is significantly increased after training compared to the situation before training (t = 3.593, *p* = 0.002, effect size of Cohen’s d = 1.532), however, *NE*_loc_ in the parietal, temporal and occipital lobes are significantly decreased (parietal and temporal lobes, t = 2.342, *p* = 0.027, effect size of Cohen’s d = 0.885; occipital lobe, t = 4.413, *p* = 0.002, effect size of Cohen’s d = 2.791). A similar phenomenon is found in the Snake game task, with Figure 14B showing that *NE*_loc_ in the parietal, temporal, and occipital lobes are significantly reduced after training compared with the pre-training condition (parietal and temporal lobes, t = 5.765, *p* < 0.001, effect size of Cohen’s d = 2.179; occipital lobe, t = 2.637, *p* = 0.030, effect size of Cohen’s d = 1.668). It shows that after the subjects undergo cognitive training intervention, the efficiency of parallel information transmission in the network of nodes in the frontal lobe is improved. This may be related to the fact that the frontal lobe area is responsible for reasoning, calculation, motor control and problem solving (Ming, 2018), which proves that the training of the operation task promotes the improvement of the subjects’ operation control ability and task-related auxiliary ability. Conversely, nodes in the parietal, temporal, and occipital lobes are less efficient in parallel information transfer in the network. In addition, when EEG was at high frequency, the difference between *NE*_loc_ before and after cognitive training was not significant, the reason may be that the connectivity properties of functional brain network of high frequency EEG are mainly task-dominated, which further confirms the above inference.

In conclusion, we use Table 2 to summarize the results of the mental state evoked experiment and the cognitive training intervention experiment.

**TABLE 2:** Overview of experimental results.

	Categories	Results summary
Mental state evoked experiment	Fatigue	The functional brain network connection density increased in fatigue state, and its small-world network parameters σ and γ were significantly reduced. It shows that the “economic” of the brain network in a fatigued state decreases, the information processing and transmission ability declines, and the human brain needs to consume more resources to maintain the level of perception and cognition.
	Attentive state	The σ in the attentive state increased significantly, indicating that maintaining concentration during work can improve the “economy” of the brain network, the utilization of brain resources is more efficient, and the allocation of resources is more concentrated. In addition, the *NE*_loc_ in the occipital lobe area also increased significantly in the attentive state, which proved that the occipital lobe area played a positive role in improving the subjects’ attention.
	Positive and negative states	Functional brain networks in positive and negative states exhibit asymmetry and differences between the left and right hemispheres. The significant increase in σ and γ in the positive state indicates that the “economic” of the brain network has improved, and the information processing and transmission capabilities have become stronger, so that the human brain can more efficiently maintain the level of perception and cognition required for work.
	Common characteristics	The functional brain networks of typical mental states during work and non-work differ in their characteristics.
Cognitive training intervention experiment	Before and after training	No significant differences were found in global properties of functional brain networks before and after training, but significant differences occurred in local properties. For example, the *NE*_loc_ in the frontal lobe region was significantly increased after training, however, the *NE*_loc_ in the parietal, temporal, and occipital lobes were significantly decreased, indicating that the efficiency of parallel information transmission in the network by nodes in the brain region has changed.

## Discussions

In this paper, the dynamic changes of perception, cognitive level and operational skills are mainly attributed to the diversity of mental states and neuroplasticity of the brain, and the phenomenon was innovatively explained from the two dimensions of brain space and time. A typical mental state evoked paradigm and cognitive training intervention paradigm were designed, and a functional brain network analysis method was developed. It was revealed that mental state and neuroplasticity during work can change the information processing efficiency and the response mechanism of brain regions, resulting in changes in perception, cognitive level and operational skills. This provides a theoretical basis for studying the relationship between neural information processing and behavior.

### Changes in mental state during work

Firstly, by setting the task mode and applying stimulation conditions, various mental states will be induced during work, and there is a dynamic transformation between each mental state. Secondly, there is a clear difference between the mental state during work and the mental state during non-work. For example, compared with the resting state, the connection density of the functional brain network of high-frequency EEG did not decrease but increases in the fatigue state during work. The reason is that the subject needs to mobilize more brain resources to maintain a high level of perception and cognition to perform the task. Thirdly, the information of the mental state induced by the task is mainly contained in the low-frequency components of the EEG, and the high-frequency components of the EEG mainly contain task-related information. For example, when comparing resting and fatigued states, attentive and inattentive states, and positive and negative states, significant differences were found in the functional brain networks of low-frequency EEG that were altered by changes in mental state. However, brain networks with high frequency EEG had similar local connectivity features that were not influenced by mental state but were influenced by task. Finally, the mental states induced by work are more complex than those induced by non-work. From the analysis of functional brain network, it was found that the characteristic of opposition between the typical oppositional mental states (such as attentive and inattentive states, positive and negative states) during work is not obvious. The reason is that the degree and perspective of the mental state induced during work and non-work (such as audio, video and other image-induced paradigms or imagination-induced paradigms) are different. Furthermore, the human brain needs to perform corresponding memory, decision-making and limb control tasks during work, so the brain needs to regulate resources to maintain a high level of perception and cognition. Therefore, the collected EEG not only contains mental state information, but also contains information about performing tasks.

### Changes in neuroplasticity during work

From the perspective of neurobiological research, some researchers pointed out that neuroplasticity mainly includes three types: (1) Synaptic plasticity refers to the strengthening or weakening of synapses that promote the transmission of electrochemical signals between neurons. Changes in synapses may be caused by changes in the concentration of neurotransmitter molecules in synapses and may also be attributed to changes in post-synaptic receptor conduction (Citri and Malenka, 2008). Neurobiological studies have pointed out that the whole life course of synapses will be constantly adjusted and reconstructed to meet the needs of body function due to the changes of internal and external environment. The regulation of synaptic connection strength is controlled by synaptic plasticity in the nervous system, which regulates the synaptic connection strength through the activation state of pre-synaptic and post-synaptic neurons. (2) Intrinsic plasticity refers to the changes in the inner ability of neurons to generate or propagate action potentials. It happens inside neurons rather than at a single synapse between them. The intrinsic plasticity of neurons is usually used to self-regulate neuronal activity, accompanied by a homeostasis mechanism to maintain the dynamic balance of neuronal firing activity (Brown and Randall, 2009). And unlike the regulation of synaptic strength, individual neurons can also change their internal excitability to adapt to different synaptic inputs by changing voltage-gated ion channels (Li et al., 2013). (3) Structural plasticity is associated with the growth and development of synapses and affects the formation and destruction of synapses. structural plasticity observed in serial section electron microscopy is accompanied by synaptic deletion and geometric transformation of the dendritic spine, and that synapses with smaller spine heads are more likely to be deleted (Bourne and Harris, 2011). In fact, in most areas of the human brain, there is a large, sparse, dynamic structure of connections between neurons that generates many potential neural circuits and helps enhance the system’s robustness to adapt to changing synaptic inputs (Cui et al., 2017). Changes in neural circuitry regulated by structural plasticity also greatly extend the memory storage capacity of the nervous system to adapt to changing internal and external environments (Berry and Nedivi, 2016). From a data-driven research perspective, we found significant differences in functional brain networks before and after cognitive training. For example, after subjects underwent cognitive training intervention, nodes in the frontal lobe increased the efficiency of parallel information transmission in the network, whereas nodes in the parietal, temporal, and occipital lobes decreased. Accordingly, we propose two hypotheses, the first one is the task relevance hypothesis: the parietal lobe is associated with touch, pressure, temperature, and pain; the temporal lobe is associated with perception, auditory stimulus recognition, and memory; and the occipital lobe is associated with vision (Ming, 2018). Because there are some brain regions whose function is not strongly related to the game task, while others are more strongly related. Therefore, the brain will regulate the distribution of information flow and resources in the whole brain, resulting in a decrease in the activity of nodes with weaker associated regions in the network, thereby reducing the *NE*_loc_ of the associated regions. The second is the brain resource requirement hypothesis: after cognitive training intervention, when the brain maintains the same level of perception, cognition and limb control, the need for information processing capacity and brain resources is reduced. For example, the subjects played the game for many times, and their sensitivity to a certain important image or sound stimulus in the game would increase with the proficiency of the game task, and the reaction time would become shorter and shorter. The required information flow and brain resources are also less and less, resulting in lower *NE*_loc_ in the brain regions responsible for the corresponding functions. Through the research on local attributes, it was found that after cognitive training intervention, the brain adjusted the distribution of information flow and resources in the whole brain. The node information processing and transmission capabilities of some brain regions are improved, while others are relatively reduced, which improves the efficiency and economy of functional brain networks as a whole. This further suggests that interventions through cognitive training can induce remodeling of the global topology in cortical connectivity networks and altered neuroplasticity. The findings of this paper echo with the research on neuroplasticity in neurobiological perspectives.

No matter whether the time span is years, months, days, hours, or minutes, in the process of evolution, development, and remodeling of living organisms, The connection strength of synapses between neurons, the intrinsic activation of neurons and the physical structure are all shaped by the changing internal and external environment all the time. Some scholars have proved this phenomenon through experiments. For example, Romero et al. (2008) designed a cognitive training intervention experiment on memory and reasoning. The experiment lasted for about 1 week. And the study found that the subjects had neuroplastic changes, which were reflected in the characteristics of EEG and event-related potentials. Wynn et al. (2019) designed a high-frequency visual stimulation paradigm to induce neuroplastic changes in visual evoked potential components. The experiment lasted for 2 weeks and proved that schizophrenia patients have neuroplastic deficits compared to normal people (Wynn et al., 2019). Seppanen et al. (2012) study found that long-term trained adult musicians have faster auditory perceptual learning than normal people, and music training also modulates rapid neural plasticity for sound encoding. The time span of the cognitive training intervention paradigm we designed is at the hour level, and the designed cognitive training task focuses on the subjects’ brain response ability, limb manipulation ability, memory and reasoning ability, and the purpose is to study the neuroplastic changes of users during work. Compared with previous studies, our study fills the gaps in related fields to a certain extent. The purpose of this paper is to demonstrate whether cognitive training interventions during work can induce neuroplastic changes in operators. In the next step, we will further study the effect of cognitive training intervention duration on the degree of changes in user neuroplasticity. By designing an experimental paradigm with a time span of hours, days, weeks, and months, we will study the degree of neuroplasticity and the types of neuroplastic changes that occur in the subjects.

### Coupling properties of mental state and neuroplasticity

We attribute the two main reasons for the changes in user perception, cognitive level, and operational skills to changes in mental state diversity and neuroplasticity during work. Because the topology of the functional brain networks underlying each mental state varies significantly in spatial dimensions and is diverse, we classify it as a property of the brain spatial dimension. Since neuroplasticity is a change in brain neural activity and neumorphism that requires repeated and regular reinforcement training in the time dimension, we classify it as a characteristic of the brain time dimension. For the purpose of engineering application, we systematically reveal the changes of mental state and neuroplasticity of the brain induced by work, these two human factors will change the response mechanism of brain regions, the efficiency of information processing and the allocation of brain resources, thereby affecting the user’s perception, cognitive level and operational skills. Moreover, most of them appear at the same time, and they accompany and affect each other. For example, professional drivers (or professional operators of special equipment) will experience neuroplastic changes in their brains compared to novices due to long-term and regular training, making their operating skills far superior to novices. When encountering emergencies in the operation, the mental state caused by them will also be different. Novices often have tension and stress reactions, while professional drivers are relatively calm. Therefore, it is necessary to carry out research on the interaction and coupling of mental state and neuroplasticity of the brain during work in the future. When addressing the impact of human factors on human-machine collaboration systems, these two factors are best considered together rather than separately. And it is not only necessary to consider the respective characteristics of these two factors, but also the coupling effect between them should be comprehensively considered.

### Future work and limitations

The brain is a complex network consisting of spatially distributed regions dedicated to different functions. It is proposed that cognitive functions emerge from dynamic interactions of several brain areas, not from the activation of a single brain region. We propose here that the brain connectome can be used not only to characterize the diversity of mental states and changes in neuroplasticity, but also to analyze the information processing mechanisms and mental expression mechanisms in the brain, so as to understand the reasons for the changes in perception and cognition levels and the underlying neurobiological mechanisms in the process of human-machine collaboration. This research contributes to the improvement of human-computer interface and the development of new adaptive automation systems. And it helps to drive a new mode of human-machine interaction. This model is trying to transform the way humans and machines work together. It integrates neurophysiological detection techniques in evaluating the performance of human-machine systems, rather than relying solely on the measurement of workers’ explicit behaviors and subjective perceptions. At the heart of this model lies the first grasp of how the brain processes perceptual and cognitive information. Then, through the monitoring, learning and inference of continuous neurophysiological signals, we can understand some changing trends, behavior patterns and application scenarios related to the user’s job content and goals, so as to be more conducive to application in complex and dynamic human-machine interaction environment. Based on the research conclusions of this paper, we will carry out deeper research in the future, and evaluate the quality and performance of system work information processing by analyzing the perception and cognition level of operators on work tasks and the functional neural network behind them, and compare it with the actual work performance, and then prompt or interfere with the operator to improve the operation or adaptively adjust the system work parameters to improve the system work performance and ensure the safety of personnel and the best performance. Additionally, we will develop brain augmentation systems from a human-centric perspective by dissecting the laws of mental state diversity and neuroplasticity that occur during human-machine interaction. This mainly includes neuro-enhancement technology and augmented cognition technology. Among them, the goal of neuro-enhancement technology is to use current advanced stimulation methods (such as transcranial magnetic stimulation and transcranial direct current stimulation) to enhance people’s visual, auditory, sustained attention, positive emotion retention, working memory, logical reasoning, and motor learning ability. The goal of augmented cognition technology is to start from the known limitations of human cognition and use computer-based methods to design to break through the bottleneck of human beings and solve the deviations and deficiencies in human cognition.

On the basis of the research conclusions of this paper, in order to pursue deeper research, the influence of individual and task differences on mental state and neuroplasticity can be the content of further research. For example, in the aspect of neuroplasticity, whether the changes of neuroplasticity have an important relationship with the cognitive level and behavioral ability of the subjects. If the cognitive training task is too difficult or too easy for the subjects, whether it will make it difficult for them to maintain motivation and attention. Such as some people may be exhausted by excessive cognitive workload, while others may be bored by too simplistic cognitive workload. Whether these two extremes negatively affect the user’s neuroplasticity. These studies can further explain why functional brain networks in some cases did not differ significantly. Therefore, the next step is to pay attention to the differences of individual users in the human-machine collaboration systems, which needs to be studied urgently.

Despite the significance of this work, several limitations should be considered. First of all, the human factors studied in this paper mainly include mental state and neuroplasticity. It has to be admitted that human factors include many aspects. It is a comprehensive definition, and many human-related factors can be classified into it, and there are also many explanations and descriptions (Wang et al., 2020), and so far there is no accepted name or definition in academia. But this does not affect some of the contributions made by this paper in related fields such as human-machine collaboration, human factors engineering, and neuroergonomics. Secondly, it has to be admitted that compared with MEG and fMRI, EEG has the disadvantage of low spatial resolution, which results in that the spatial resolution of functional brain networks established by EEG cannot be accurate to very fine areas. However, considering its high temporal resolution and the portability of EEG acquisition equipment, it provides feasibility for the study of mental state diversity and neuroplasticity of the brain during work, which is why we choose EEG. Finally, a total of 26 subjects participated in the experiment, which to a certain extent can prove the correctness of the proposed ideas and highlight some important experimental results. Further research will involve testing the proposed hypotheses and results with a larger number of subjects and expanding the research to include studies of inter-individual and gender differences.

## Conclusion

In this paper, the evoked paradigm of typical mental state and the analysis method of its functional brain network were proposed, and it was revealed that the mental state of the subjects would change dynamically during the work process and showed the characteristics of diversity. There were significant differences between functional brain networks in different mental states, and the information processing efficiency and the mechanism of brain area response had changed significantly. At the same time, the cognitive training intervention paradigm and its functional brain network analysis method were proposed. It revealed that there was a significant difference between the functional brain networks of the subjects before and after the cognitive training intervention experiment, and the brain adjusted the distribution of information flow and resources in the whole brain. The node information processing and transmission capabilities of some brain regions are improved, while others are relatively reduced, which improves the efficiency and economy of functional brain networks as a whole. This further suggests that interventions through cognitive training can induce remodeling of the global topology in cortical connectivity networks and altered neuroplasticity. To sum up, this paper innovatively and comprehensively studies the dynamic changes of the brain in space and time dimensions, and reveals that mental state and neuroplasticity during work can change the information processing efficiency and the response mechanism of brain regions, resulting in changes in perception and cognitive levels. We expound the complexity of human factors in the field of human-machine interaction and the value behind them from a more comprehensive dimension, which can effectively improve the ability of humans to understand and simulate complex human-machine system interactions at work. The conclusion of this study provides a theoretical basis for the relationship between neural information processing and behavior, and enriches the research connotation in the field of neuroergonomics.

## Data availability statement

The raw data supporting the conclusions of this article will be made available by the authors, without undue reservation.

## Ethics statement

The studies involving human participants were reviewed and approved by Institutional Review Board of Xi’an Jiaotong University. The patients/participants provided their written informed consent to participate in this study. Written informed consent was obtained from the individual(s) for the publication of any potentially identifiable images or data included in this article.

## Author contributions

TZ proposed and did the research and wrote the manuscript. XZ proposed the research idea, supervised the work, and revised the manuscript. WZ organized and conducted part of the experiment. ZL assisted in processing experimental data. YW assisted in collecting experimental data. YZ revised the manuscript. All authors contributed to the article and approved the submitted version.
